# [^99m^Tc]Tc-antigranulocyte scintigraphy for prediction of bone marrow reserve prior to radioligand therapy in patients with metastatic castration resistant prostate cancer

**DOI:** 10.1007/s00259-025-07319-7

**Published:** 2025-05-06

**Authors:** Sophie Carina Kunte, Astrid Delker, Adrien Holzgreve, Mathias J. Zacherl, Maximilian Scheifele, Jozefina Casuscelli, Franz Josef Gildehaus, Marcus Unterrainer, Harun Ilhan, Rudolf A. Werner, Lena M. Unterrainer

**Affiliations:** 1 Department of Nuclear Medicine, LMU University Hospital, LMU Munich, Marchioninistr. 15, 81377 Munich, Germany; 2 Bavarian Cancer Research Center (BZKF), Partner Site, Munich, Germany; 3 Ahmanson Translational Theranostics Division, David Geffen School of Medicine at UCLA, Los Angeles, CA USA; 4 Department of Urology, LMU University Hospital, LMU Munich, Munich, Germany; 5 Die Radiologie, Munich, Germany; 6 Division of Nuclear Medicine, The Russell H Morgan, Department of Radiology and Radiological Sciences, Johns Hopkins School of Medicine, Baltimore, USA

**Keywords:** [^99m^Tc]Tc-antigranulocyte scintigraphy, PSMA PET/CT, PSMA RLT, Indication, Pancytopenia, Myelotoxicity

## Abstract

**Purpose:**

Radioligand therapy (RLT) targeting PSMA (prostate-specific membrane antigen) has transformed the treatment of metastatic castration-resistant prostate cancer (mCRPC). However, bone marrow depletion remains a common side effect, particularly in patients with extensive bone metastases or prior myelotoxic therapies. This study evaluated [^99m^Tc]Tc-antigranulocyte scintigraphy to assess bone marrow reserve to guide myelotoxic treatment decisions.

**Methods:**

Ten mCRPC patients with extensive osseous tumor load on [^18^F]F-PSMA PET/CT underwent [^99m^Tc]Tc-antigranulocyte scintigraphy to assess bone marrow reserve and RLT eligibility (interval: 26.6 ± 18.5 days). Visual comparison of both modalities evaluated tumor-bone marrow overlap. Patients without significant co-localization received RLT ([^177^Lu]Lu-PSMA or [^225^Ac]Ac-PSMA) with laboratory monitoring before and between the cycles.

**Results:**

No significant co-localization between viable bone marrow and PSMA-positive metastases was observed in 9/10 (90.0%) patients. Two other patients were excluded from RLT due to contraindications. Of the remaining seven patients, 2/7 (28.6%) underwent subsequent [^177^Lu]Lu-PSMA therapy, 2/7 (28.6%) [^225^Ac]Ac-PSMA therapy and 2/7 (28.6%) [^225^Ac]Ac-[^177^Lu]Lu-PSMA therapy. 1/7 (14.3%) received a single cycle of [^177^Lu]Lu-PSMA, which was then followed by [^225^Ac]Ac-PSMA therapy. Follow-up laboratory results showed no significant changes from baseline (*p* > 0.05).

**Conclusion:**

[^99m^Tc]Tc-antigranulocyte scintigraphy may support the assessment of bone marrow reserve in patients with extensive bone metastases. Spatial mismatch was associated with no significant myelotoxicity, whereas high congruence may increase the risk. No fixed thresholds currently exist; thus, this method should be regarded as a supplementary tool in complex cases and future studies are needed to define the critical bone marrow volume.

## Background

The treatment of metastatic castration-resistant prostate cancer (mCRPC) often remains challenging. However, radioligand-therapy (RLT) targeting the surface protein PSMA of prostate cancer cells has revolutionized treatment options [1]. The results of recently published clinical trials have demonstrated a significantly prolonged overall survival rate in patients treated with the radiopharmaceutical [^177^Lu]Lu-PSMA compared to the standard of care [2, 3]. This has led to the official approval [^177^Lu]Lu-PSMA in patients with mCRPC by the Food and Drug Administration (FDA) and European Medicines Agency (EMA) [3, 4]. Another novel radioligand that overcomes the [^177^Lu]Lu-PSMA -associated radioresistance is [^225^Ac]Ac-PSMA, showing promising results [5–7]. However, a common side effect of RLT is the depletion of bone marrow stem cell reserve. Especially, patients with extensive osseous tumor load or former myelotoxic treatment (e.g. chemotherapy or prior RLT) are at risk [8, 9]. Osseous tumor burden can be assessed by PSMA PET/CT; however, bone marrow function is usually assessed using lab samples. Whilst peripheral blood counts offer a momentary reflection of hematopoietic function, they lack spatial resolution and may fail to capture early or regionally confined marrow compromise, particularly in patients with extensive osseous metastases, prior myelotoxic therapies, or underlying bone marrow pathology. Conversely, imaging with ^99m^Tc-labelled antigranulocyte antibodies provides a whole-body assessment of myelopoiesis.

Therefore, in this case series, we assessed, if [^99m^Tc]Tc-antigranulocyte scintigraphy can be used for evaluation of bone marrow reserve in order to simplify decision making on potential myelotoxic treatments.

## Materials and methods

### Patients

All patients with mCRPC and (1) diagnosed with an extensive osseous tumor load on [^18^F]F-PSMA PET/CT, who (2) additionally underwent [^99m^Tc]Tc-antigranulocyte scintigraphy prior to RLT with either [^177^Lu]Lu-PSMA-617, [^177^Lu]Lu-PSMA I&T or [^225^Ac]Ac-PSMA I&T at the Department of Nuclear Medicine, LMU University hospital, LMU Munich were included into this retrospective study. This analysis was performed in compliance with the principles of the Declaration of Helsinki and was approved by the institutional ethics board of the LMU Munich (IRB 24–0982). General patient characteristics, laboratory analyses (baseline and follow-up) as well as imaging data were collected and compiled in an anonymized data sheet.

### Radiopharmaceutical and imaging protocol

[^99m^Tc]Tc-antigranulocyte (Scintimun^®^) scintigraphy and [^18^F]F-PSMA PET/CT were conducted at a mean interval of 26.6 ± 18.5 days.

The ^99m^Tc-labelled monoclonal antibody BW 250/183 (Scintimun^®^, CIS Bio International, Gif-sur-Yvette Cedex, France) was prepared according to the manufacturer’s instructions and injected intravenously at a mean activity of 677.8 ± 141.4 MBq. Whole body planar scintigraphies including anterior and posterior views of the whole body were acquired 4 h after tracer injection. Scintigraphies including a low-doses SPECT/CT were acquired at the Department of Nuclear Medicine, LMU Munich using a Siemens Symbia T (Siemens Medical Systems, Hoffman Estates, IL, USA) dual-head, large field of view camera with high-resolution, low-energy collimators and acquisition speeds of 30 cm min^− 1^, 12 cm min^− 1^ and 5 cm min^− 1^. Images were reconstructed using a dedicated software package (Hermes hybrid viewer, Hermes Medical Solutions, Stockholm, Sweden).

[^18^F]F-PSMA-1007 radiotracer was provided as an in-house production at the Department of Nuclear Medicine, LMU Munich, Germany and the radiosynthesis was performed as described earlier [10, 11].

Quality control measurements were in accordance with local product release criteria. [^18^F]F-PSMA-1007 was injected intravenously (mean activity 272.0 ± 34.8 MBq). All PET/CT scans were acquired at the Department of Nuclear Medicine, LMU Munich using a Siemens Biograph mCT flow (Siemens Healthineers, Erlangen, Germany) or Siemens Biograph 64. Scans were acquired 60 min after tracer injection [10]. If no medical contraindication was given, patients received furosemide as a premedication for radiation protection and 1.5 mL of iopromide (Ultravist-300, Bayer Healthcare, Leverkusen, Germany) per kg body weight to obtain contrast-enhanced, diagnostic CT scans in portal-venous phase [12]. Images were reconstructed iteratively using TrueX (3 iterations and 21 subsets, 3D Gauss post-filter of 4-mm full width half maximum). Slice thickness on CT was 0.3 cm.

### Image analysis

A dedicated software package was used (Hermes hybrid viewer, Hermes Medical Solutions, Stockholm, Sweden).

The PET/CT scan was analyzed with consideration of the extent of the osseous tumor. Bone marrow reserve was determined using scintigraphy.

The two modalities were then compared visually, in order to determine whether the extent of skeletal metastases compromised bone marrow reserve. Therefore, the spatial relationship between the osseous tumor burden and the bone marrow distribution was assessed, as follows:

#### Step 1: identification of bony tumor burden

The PSMA-PET/CT was used to delineate the extent of bone metastases. The tumor burden within the tubular bones, e.g. femur, and pelvis was mapped, focusing on regions with high radiotracer uptake, indicating active metastatic involvement.

#### Step 2: identification of bone marrow distribution

The [^99m^Tc]Tc-antigranulocyte scintigraphy was then evaluated to identify regions of active hematopoietic bone marrow. Particular attention was paid to the pelvis, femurs and vertebrae as these are key sites for hematopoiesis.

#### Step 3: Spatial overlay analysis

The images from both modalities were visually aligned and compared side-by-side to assess the degree of spatial overlap between the metastatic lesions and areas of active bone marrow. Two primary scenarios were identified: (1) High spatial overlap, if the majority of active bone marrow areas overlapped with metastatic lesions, the patient was considered to have insufficient bone marrow reserve and thus, considered ineligible for RLT due to the increased risk of hematological toxicity. (2) Low/no relevant spatial overlap, if metastatic lesions and active bone marrow regions were largely separated, it was assumed that sufficient bone marrow reserve was available. These patients were considered eligible for RLT.

### Follow up

Patients underwent a laboratory analyses of hemoglobin, leucocytes, platelets, neutrophils as well as PSA, AP and LDH prior to and about 4 weeks after every RLT cycle.

### Statistical analysis

Data analysis was performed using Microsoft Excel (Excel 2019, Microsoft, Redmond, WA, USA) and GraphPad Prism (Version 9.5.0 (730)). Descriptive statistics are displayed as mean ± standard deviation (SD). Shapiro-Wilk normality test was performed. Group comparisons were performed using a parametric or non-parametric paired t-test. A two-tailed p-value < 0.05 was considered statistically significant.

## Results

### [^99m^Tc]Tc-antigranulocyte scintigraphy and [^18^F]F-PSMA-PET/CT

**Fig. 1:**
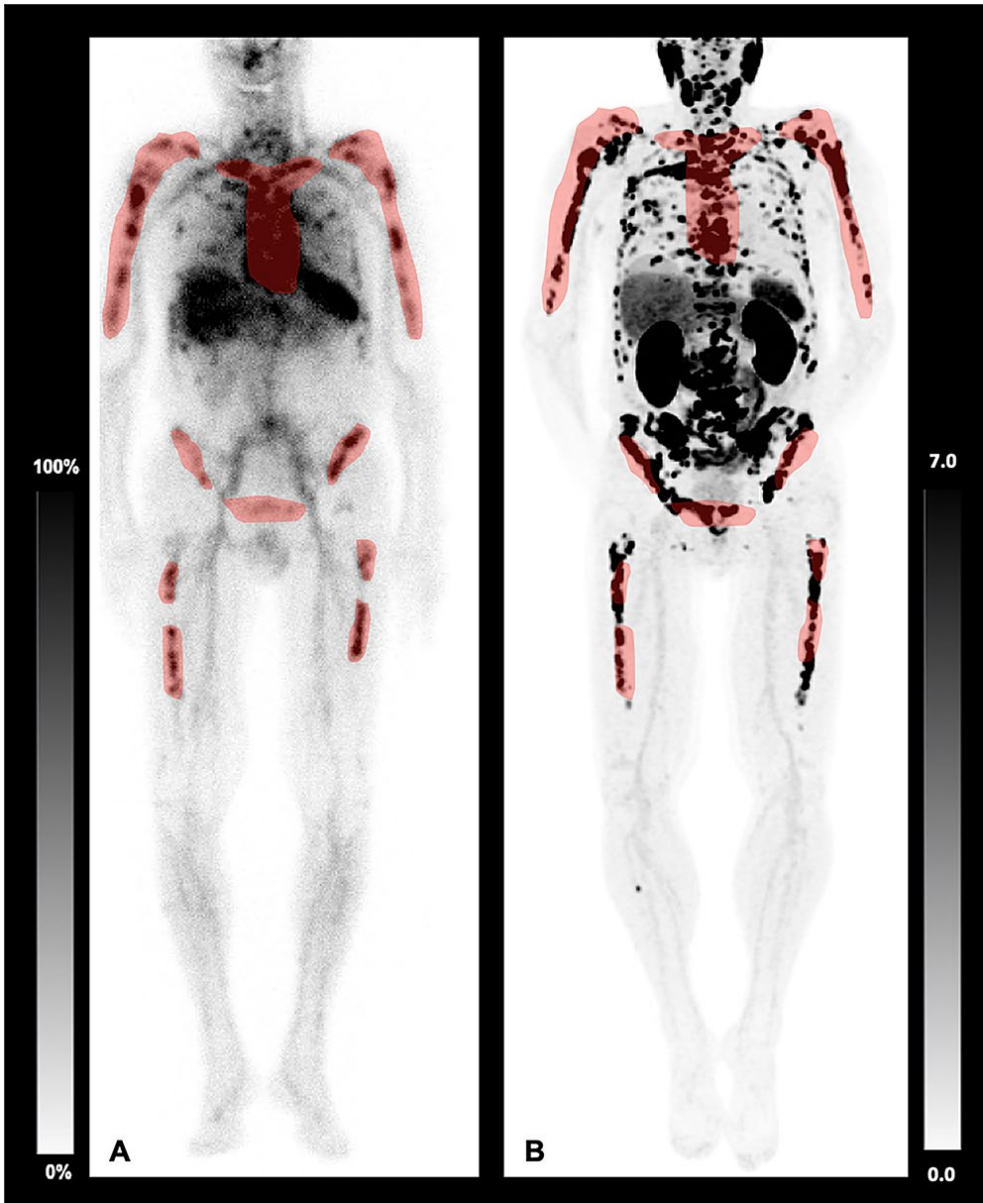
81-year-old patient prior to PSMA-RLT undergoing **A**) [^99m^Tc]Tc-antigranulocyte scintigraphy and **B**) [^18^F]F-PSMA-PET/CT presenting with congruence of viable bone marrow and osseous metastases, as demonstrated in red. Moderate bone marrow expansion is observed in the proximal extremities. **A** comparison of the results of the PSMA PET/CT scan with the other imaging techniques reveals extensive co-localization of the areas with hematopoietic bone marrow and metastatic infiltration. Nevertheless, more extensive areas of hematopoietic bone marrow that are not subject to metastatic infiltration cannot be identified. The pronounced augmentation of the spleen is in accordance with the phenomenon of extramedullary hematopoiesis

In total, 10 patients at a mean age of 75.0 years (SD 5.8) diagnosed with mCRPC and extensive osseous tumor load on [^18^F]F-PSMA PET/CT were included (Table 1). 4/10 (40.0%) presented with disseminated osseous metastases and 6/10 (60.0%) with diffuse marrow infiltration. Pretreatments are displayed in Table 1, 6/10 (60.0%) underwent chemotherapy, and 3/10 (30.0%) prior radioligand therapy. The mean time interval between PET and [^99m^Tc]Tc-antigranulocyte scintigraphy was 26.6 ± 18.5 days. Bone marrow reserve as determined by [^99m^Tc]Tc-antigranulocyte scintigraphy was visually reduced in 8/10 (80.0%) patients (Fig. 1). Of note, one patient who did not have reduced bone marrow reserve had received prior chemotherapy, and the other had not received prior myelotoxic treatments.

**Table 1:** Patient characteristics

ID	Age	Pretreatment	Eligible for RLT in regard of overlap	Other contraindication	Total activity Lu-PSMA [GBq]	Total activity Ac-PSMA [MBq]
1	72.5	ADT, ARSI, Docetaxel	yes	midgut bleeding		
2	69.9	ADT, ARSI, Lutetium, RT	yes		12.2	32.5
3	71.8	ADT, RT, Docetaxel	yes		12.2	
4	80.3	ADT, Docetaxel	yes		6	7.9
5	75.8	RT	yes	infection		
6	83.0	ADT, ARSI, RT, Lutetium	yes		30.2	
7	81.3	ADT, ARSI, RT, 223-Radium	no			
8	79.7	ADT, ARSI, RT, Docetaxel, Actinium, Lutetium	yes		22.9	27.6
9	70.1	Cabazitaxel, Docetaxel, RT	yes		4.3	28
10	65.8	ADT, ARSI, Docetaxel	yes		3.3	25.2
**Mean**	**75.0**				**13.0**	**24.2**
**SD**	**5.8**				**10.1**	**9.5**

Ac Actinium, ADT androgen-deprivation therapy, ARSI androgen receptor signaling inhibitor, Lu Lutetium, PSMA prostate-specific membrane antigen, RLT radioligand therapy, RT radiation therapy

**Fig. 2:**
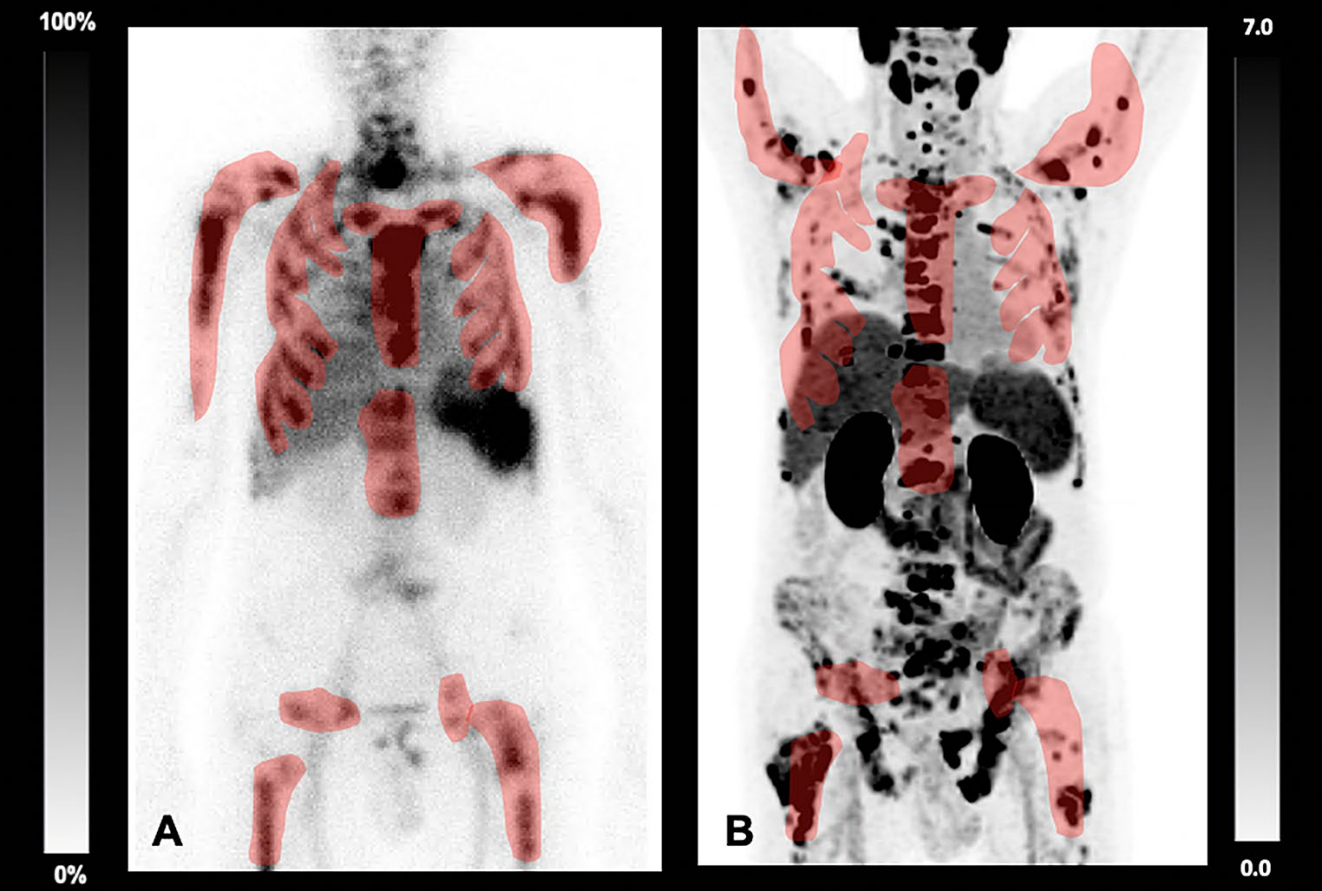
80-year-old-patient prior to PSMA-RLT undergoing **A**) [^99m^Tc]Tc-antigranulocyte scintigraphy and **B**) [^18^F]F-PSMA-PET/CT presenting with no relevant spatial overlap between viable bone marrow and osseous metastases, as demonstrated in red

1/10 (10.0%) showed a high bone marrow reserve, however, the PET scan revealed visually almost congruence of viable bone marrow and osseous metastases, thus, this patient was excluded from RLT (Fig. 2).

### Patients and change of clinical parameters

A total of nine patients were deemed eligible for RLT on the basis of visually hardly any overlapping of osseous metastases and viable bone marrow. However, two patients were excluded from RLT due to the presence of acute contraindications (severe infection and midgut bleeding). 2/7 (28.6%) patients underwent [^177^Lu]Lu-PSMA, 2/7 (28.6%) patients [^225^Ac]Ac-PSMA therapy and 2/7 (28.6%) patients [^225^Ac]Ac-[^177^Lu]Lu-PSMA-therapy. 1/7 (14.3%) patients received a single cycle of [^177^Lu]Lu-PSMA, which was then followed by [^225^Ac]Ac-PSMA therapy.

**Fig. 3:**
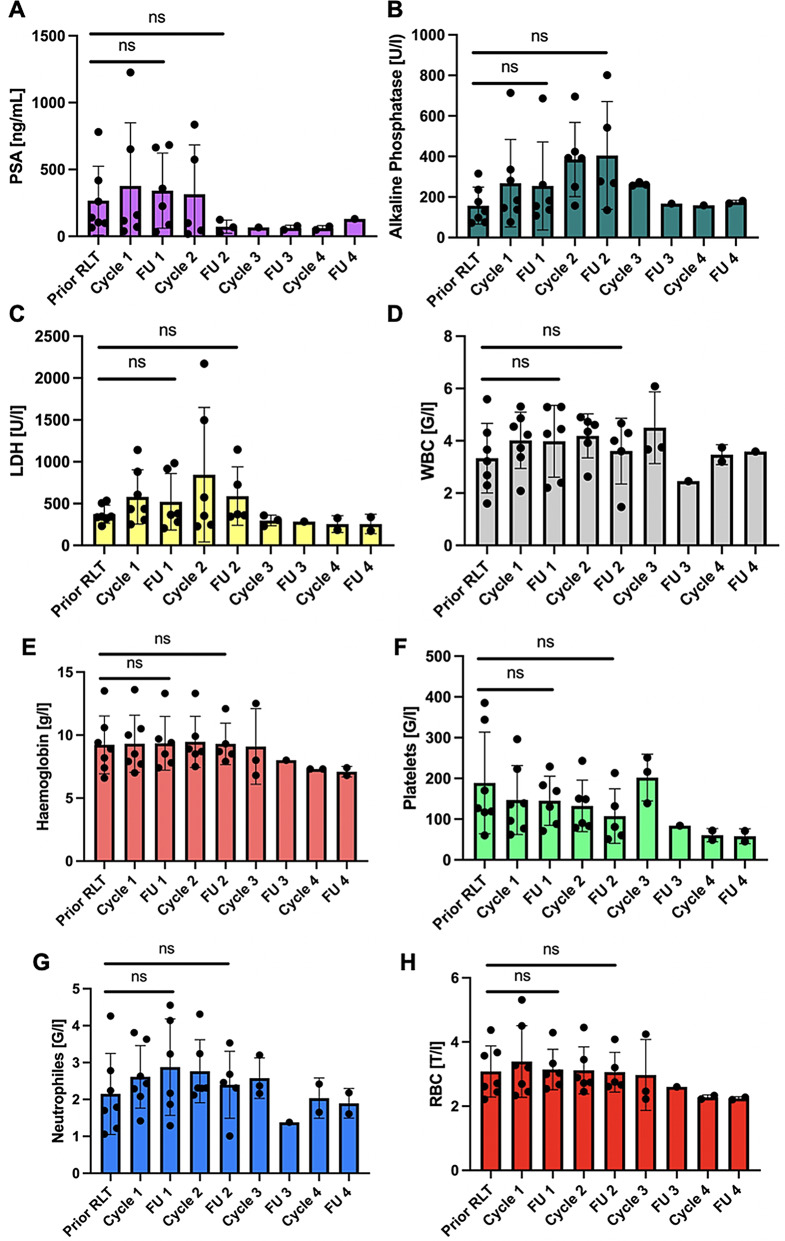
Laboratory analyses show no significant change between prior to RLT, FU 1 and FU 2, respectively. **A**) PSA (prostate-specific antigen), **B**) Alkaline phosphatase, **C**) LDH (lactate-dehydrogenase), **D**) WBC (white blood cells), **E**) Hemoglobin, **F**) Platelets, **G**) Neutrophiles, **H**) RBC (red blood cells). ns not significant

Prior to RLT, the mean results of the laboratory analyses were as follows: hemoglobin level 9.2 ± 2.3 G/dL, RBC count 3.1 ± 0.8 G/l, WBC count 3.3 ± 1.3 G/l; platelets 189 ± 125 G/l, neutrophils 2.2 ± 1.1 G/l, LDH 374 ± 108 U/l, PSA 202 ± 210 mg/mL and AP 157 ± 91 U/l (Table 2; Fig. 3).

**Table 2:** Laboratory analysis prior to RLT

Pat. ID	PSA [mg/ml]	AP [U/l]	LDH [U/l]	WBC [G/l]	Hb [G/l]	Platelets [G/l]	Neutrophiles [G/l]	RBC [G/l]
2	102	98	344	3.7	6.6	119	2.8	2.2
3	143	176	506	2.7	8.2	170	1.7	2.6
4	411	236	535	2.3	10.6	385	1.2	3.6
6	62	130	345	5.6	9.4	344	4.3	3.7
8	20	75	233	1.6	8.9	60	1.1	2.7
9	581	315	319	3.2	7.4	117	2.2	2.4
10	98	72	336	4.3	13.5	127	1.8	4.4
**Mean**	202	157	374	3.3	9.2	189	2.2	3.1
**SD**	210	91	108	1.3	2.3	125	1.1	0.8

AP alkaline phosphatase, Hb hemoglobin, LDH lactate dehydrogenase, PSA prostate specific antigen, RBC red blood cell, WBC white blood cell. Reference: AP 40–130 U/l, Hb 13.5–17.5 G/dL, LDH < 249 U/l, neutrophils 1.78–6.23 G/l, platelets 146–328 G/l, PSA < 4.4 mg/mL, RBC 4.54–5.77 G/l, WBC 3.9–9.8 G/l

At the subsequent follow-up examination, conducted following the conclusion of the initial treatment cycle, the mean hemoglobin level was 9.4 ± 2.1 G/dL, RBC count 3.1 ± 0.6 G/l, WBC count 4.0 ± 1.4 G/l, platelets 145 ± 60 G/l, neutrophils 2.9 ± 1.3 G/l, LDH 521 ± 338 U/l, PSA 377 ± 473 mg/mL and AP 255 ± 218 U/l (Table 3; Fig. 3).

**Table 3:** Laboratory analysis at FU 1

Pat. ID	PSA [mg/ml]	AP [U/l]	LDH [U/l]	WBC [G/l]	Hb [G/l]	Platelets [G/l]	Neutrophiles [G/l]	RBC [G/l]
2	117	155	280	2.2	7.4	88	1.3	2.5
3	1225	182	915	5.3	8.5	169	4.6	2.7
4	652	686	983	5.3	9.7	229	4.1	3.3
6	71	137	380	4.3	7.8	183	3.2	3.0
8	159	108	365	2.4	9.4	71	1.9	3.0
9	n.a.	n.a.	n.a.	n.a.	n.a.	n.a.	n.a.	n.a.
10	39	260	205	4.5	13.3	130	2.2	4.3
**Mean**	377	255	521	4.0	9.4	145	2.9	3.1
**SD**	473	218	338	1.4	2.1	60	1.3	0.6

AP alkaline phosphatase, Hb hemoglobin, LDH lactate dehydrogenase, n.a. not applicable, PSA prostate specific antigen, RBC red blood cell, WBC white blood cell. Reference: AP 40–130 U/l, Hb 13.5–17.5 G/dL, LDH < 249 U/l, neutrophils 1.78–6.23 G/l, platelets 146–328 G/l, PSA < 4.4 mg/mL, RBC 4.54–5.77 G/l, WBC 3.9–9.8 G/l

The laboratory analysis conducted following the second cycle (49 ± 7 days) revealed a mean hemoglobin level of 9.3 ± 1.6 G/dL, RBC count 3.1 ± 0.6 G/l, WBC count 3.6 ± 1.3 G/I, platelets 107 ± 67 G/I, neutrophils 2.4 ± 0.9 G/l, LDH 589 ± 349 U/l, PSA 314 ± 370 mg/mL and AP 405 ± 266 U/l (Table 4; Fig. 3).

**Table 4:** Laboratory analysis at FU 2

Pat. ID	PSA [mg/ml]	AP [U/l]	LDH [U/l]	WBC [G/l]	Hb [G/l]	Platelets [G/l]	Neutrophiles [G/l]	RBC [G/l]
2	44	135	371	3.6	8.5	81	2.5	2.8
3	n.a.	n.a.	n.a.	n.a.	n.a.	n.a.	n.a.	n.a.
4	836	801	1144	4.7	9.3	213	3.5	3.2
6	n.a.	n.a.	n.a.	n.a.	n.a.	n.a.	n.a.	n.a.
8	574	542	725	1.5	7.9	51	1.0	2.6
9	100	269	359	4.0	8.7	60	2.7	2.7
10	18	277	345	4.3	12.1	132	2.3	4.1
**Mean**	314	405	589	3.6	9.3	107	2.4	3.1
**SD**	370	266	349	1.3	1.6	67	0.9	0.6

AP alkaline phosphatase, Hb hemoglobin, LDH lactate dehydrogenase, n.a. not applicable, PSA prostate specific antigen, RBC red blood cell, WBC white blood cell. Reference: AP 40–130 U/l, Hb 13.5–17.5 G/dL, LDH < 249 U/l, neutrophils 1.78–6.23 G/l, platelets 146–328 G/l, PSA < 4.4 mg/mL, RBC 4.54–5.77 G/l, WBC 3.9–9.8 G/l

The statistical analysis demonstrated that there was no statistically significant change in the laboratory values (hemoglobin (*p* = 0.376; *p* = 0.276), RBC (*p* = 0.221; *p* = 0.145), WBC (*p* = 0.151; *p* = 0.159), platelets (*p* = 0.843; *p* = 0.250), neutrophils (*p* = 0.142; *p* = 0.107), LDH (*p* = 0.769; *p* > 0.999), PSA (*p* = 0.454; *p* = 0.281), AP (*p* = 0.644; *p* = 0.894)) prior to RLT and FU 1 and FU 2, respectively (Table 5; Fig. 3).

**Table 5:** Laboratory values: level of significance (p-values)

	Prior RLT − 1. FU	Prior RLT − 2. FU
PSA	0.454	0.281
AP	0.644	0.894
LDH	0.769	> 0.999
WBC	0.151	0.159
Hb	0.376	0.276
Platelets	0.843	0.250
Neutrophiles	0.142	0.107
RBC	0.221	0.145

AP alkaline phosphatase, FU follow-up, Hb hemoglobin, LDH lactate dehydrogenase, PSA prostate-specific antigen, RBC red blood cell, RLT radioligand therapy, WBC white blood cell

As only *n* = 3 patients received three cycles and *n* = 2 patients received four cycles of RLT, no further statistical analysis was conducted for these data. However, Fig. 3 illustrates a declining trend (prior RLT-FU 4) in platelet (189 ± 125 G/l; 58 ± 18.4 G/l), RBC (3.1 ± 0.8 G/l; 2.2 ± 0.1 G/l) and hemoglobin (9.2 ± 2.3 G/dL; 7.1 ± 0.4 G/dL) levels, while WBC (3.3 ± 1.3 G/L; 3.2 ± 0.5 G/l) and neutrophil (2.2 ± 1.1 G/l; 1.9 ± 0.4 G/l) levels appear to be stable. PSA levels (202 ± 210 mg/mL; 61.5 ± 18.7 mg/mL) also exhibit a decrease, whereas LDH (374 ± 108 U/l; 256 ± 116 U/l) and AP (157 ± 91 U/l; 175 ± 8.5 U/l) levels remain consistent (Fig. 3).

## Discussion

This retrospective pilot analysis is the first of its kind to assess the use of [^99m^Tc]Tc-antigranulocyte scintigraphy in order to estimate the bone marrow reserve in patients considered for PSMA RLT. A high degree of spatial overlap between osseous metastases and viable bone marrow was interpreted as a possible risk factor for hematologic toxicity. While no definitive thresholds can yet be established, this imaging approach may aid decision-making in selected cases, especially in advanced therapy lines.

In this cohort of 10 patients, one patient exhibited almost congruence of osseous metastases and viable bone marrow. In cases of high visual congruence between osseous metastases and haematopoietically active bone marrow on imaging, PSMA RLT should be considered with caution due to the potential risk of aggravating bone marrow suppression. In such cases, alternative therapeutic strategies, if available, may be more appropriate. Whilst PSMA-targeted therapies are effective in targeting metastatic prostate cancer, they can result in the accumulation of radiation in the bone marrow. The additional radiation could exacerbate hematologic toxicity. In this patient the risk for severe myelotoxicity was increased, especially since he had a history of lymphoma and earlier chemotherapy as well as 223-Radium therapy, he was excluded from RLT. It remains uncertain whether severe myelotoxicity would have been caused by RLT in the patient with congruence; however, the risk appears to be very high.

In the other nine patients viable bone marrow and osseous metastases were predominantly not co-located. Accordingly, these patients were deemed eligible for RLT. However, only 3 out of 9 patients had a sufficient bone marrow reserve as detected by scintigraphy prior to RLT. This could be explained by prior myelotoxic treatments such as chemotherapy [13]. The statistical analysis demonstrated no statistically significant change in the laboratory values prior to RLT and FU 1 and FU 2, respectively. Despite the absence of a statistically significant myelosuppressive effect, the results suggest that [^99m^Tc]Tc-antigranulocyte scintigraphy may serve as a tool for risk assessment of myelotoxicity.

It was also observed that thrombocytopenia developed in some patients, despite no spatial overlap in both scans. However, this finding did not reach statistical significance due to the limited number of patients and the absence of multiple doses in all subjects. This prompts the question of whether [^99m^Tc]Tc-antigranulocyte scintigraphy has the potential to serve as a predictor of how many RLT cycles a patient may tolerate and might be thus suitable as an indirect marker for treatment success.

A decline of stem cell reserve during RLT might be also attributable to tumor progression (no response to therapy), especially in case of an increase or growth and further infiltration of osseous metastases. However, Kind et al. reported, that an impairment of bone marrow function in early RLT cycles is rather associated with osseous tumor progression than with myelotoxicity caused by RLT [14]. Our data support this, since AP and PSA levels remain mostly consistent, thus, there is no suspicion of biochemical progression.

To the best of our knowledge, this is the first study investigating the role of [^99m^Tc]Tc-antigranulocyte scintigraphy to assess bone marrow reserve prior to PSMA RLT. However, other studies reported an acceptable safety profile of PSMA RLT in patients with diffuse bone marrow infiltration on a clinical point of view [8, 15]. Nevertheless, studies investigating myelotoxicity through bone marrow dosimetry in patients with extensive osseous metastases are lacking.

This approach is particularly useful for strongly pretreated patients, such as chemotherapy or previous cycles of radioligand therapy, as these treatments can impact bone marrow reserve [16]. By assessing the spatial overlap between bone metastases and the hematopoietic bone marrow, this method provides a tool for assessing bone marrow function prior to initiating PSMA therapy. It also helps to guide decisions regarding continuation of RLT or even in a “rechallenge” scenario, helping to balance therapeutic efficacy with the risk of myelotoxicity.

The retrospective analysis is constrained by the limited number of patients included in the study, as well as its retrospective design. Moreover, the analysis of statistical effects was limited to up to two cycles, as the number of data points was insufficient for three cycles or more. Further investigation is required to ascertain the long-term effects, as well as any potential effects on the bone marrow, following several cycles of RLT. The heterogeneity of RLT regimens within the cohort, including differences in used radioligands, number of therapy cycles, and prior systemic treatments, is acknowledged as a potential limitation, since these factors may influence hematological outcomes. Nevertheless, the primary objective of this investigation was to establish a proof-of-concept for the use of [^99m^Tc]Tc-antigranulocyte scintigraphy in evaluating bone marrow reserve, thus the study was not designed to yield definitive clinical thresholds or treatment algorithms, but rather to demonstrate feasibility and highlight potential clinical utility.

A limitation of this study is that the one patient with a high spatial overlap was excluded from RLT due to the high risk of myelotoxicity, preventing a direct comparison of outcomes between high and low overlap groups. Consequently, the definitive benefit of scintigraphy remains uncertain, although it serves as a valuable tool for treatment decision-making. Moreover, studies with larger cohorts and subgroup analysis for the different RLT regimens are planned. It would have been desirable to quantify the amount of metastasis and bone marrow overlap. The utilization of the Dice coefficient for the analysis of the exact overlap between planar scintigraphy and PET imaging is rendered unfeasible by virtue of the fundamental disparities in the spatial resolution and acquisition methodologies of these imaging modalities. Planar scintigraphy provides a two-dimensional projection of the radiotracer distribution, with limited depth resolution and lower spatial accuracy, whereas PET imaging offers higher spatial resolution and three-dimensional information. The Dice coefficient is unable to account for due to its assumption of comparable spatial alignment and resolution. Furthermore, the inherent differences in tissue penetration, sensitivity, and signal-to-noise ratios between the two modalities further complicate direct overlap analysis. Thus, images were only analyzed visually, which might lead to a reader bias. Further publications have to implement a quantitative marker to exactly determine the differences in the tracer uptake between both imaging modalities. In this cohort there were only images with clear congruence and incongruence. Consequently, no data or even threshold of overlaps between [^99m^Tc]Tc-antigranulocyte scintigraphy and PSMA PET, where PSMA RLT might be feasible or not, can be reported. Thus, a fundamental objective for subsequent research endeavors will be the quantification of the critical volume of haematopoietically active bone marrow. Determining a cut-off value would provide clinicians with a more objective parameter to evaluate bone marrow reserve and guide individualized treatment decisions. Additionally, further studies are required to determine the bone marrow doses in these patients, with particular attention paid to dosimetry.

This study proposed [^99m^Tc]Tc-antigranulocyte scintigraphy as a clinical consideration in order to facilitate the decision-making process prior to RLT in scenarios where there is ambiguity regarding the potential for myelotoxicity. Further studies are essential to clarify the relationship between bone marrow distribution patterns, functional reserve, and clinical outcomes following PSMA RLT.

## Conclusion

In conclusion, [^99m^Tc]Tc-antigranulocyte scintigraphy appears to be a valuable tool for the assessment of bone marrow reserve in patients at risk of myelotoxicity during PSMA RLT. This imaging-based approach can help to streamline the decision-making process, particularly in complex cases where bone marrow function is compromised or uncertain due to previous treatments or underlying conditions.

## Data Availability

The datasets used and/or analyzed during the current study are available from the corresponding author on reasonable request.

## Abbreviations

Ac: Actinium
ADT: Androgen deprivation therapy
ARSI: Androgen receptor signaling inhibitor
AP: Alkaline phosphatase
FU: Follow-up
Hb: Haemoglobin
Lu: Lutetium
LDH: Lactate dehydrogenase
mCRPC: Metastatic castration-resistant prostate cancer
n.a.: Not applicable
ns: Not significant
PSA: Prostate-specific antigen
PSMA: Prostate-specific membrane antigen
RBC: Red blood cells
RLT: Radioligand therapy
RT: Radiation therapy
WBC: White blood cells
